# The Effectiveness of Personalized Bowel Preparation Using a Smartphone Camera Application: A Randomized Pilot Study

**DOI:** 10.1155/2017/4898914

**Published:** 2017-08-08

**Authors:** Jae won Jung, Jongha Park, Gi Jung Jeon, Young Soo Moon, Sung Yuon Yang, Tae Oh Kim, Eui Tay Jung, Hee-Cheol Kim

**Affiliations:** ^1^Division of Gastroenterology, Department of Internal Medicine, Haeundae Paik Hospital, Busan, Republic of Korea; ^2^College of Medicine, Inje University, Gimhae, Republic of Korea; ^3^Department of Gastroenterology, Dongnam Institute of Radiological and Medical Sciences, Busan, Republic of Korea; ^4^College of Design, Inje University, Gimhae, Republic of Korea; ^5^Department of Computer Engineering, Inje University, Gimhae, Republic of Korea

## Abstract

**Background:**

We aimed to investigate the effectiveness of a smartphone application that analyzes and judges the optimal dosage of polyethylene glycol (PEG) for bowel preparation.

**Methods:**

Patients were assigned to use the smartphone camera application (app group) or written instructions (non-app group). The smartphone camera application was programmed to analyze the bowel preparation quality and automatically determine the dosage of PEG from an analysis of stool images. In contrast, the non-app group consumed PEG solution according to the manual.

**Results:**

The primary outcome was the quality of the bowel preparation based on blinded ratings using the Ottawa bowel preparation scale (OBPS). There was no statistically significant difference in the mean OBPS scores between the two groups (*P* = 0.950). However, the app group consumed a lower dose of PEG than the non-app group (mean dosage (mL): 3713.2 ± 405.8 versus 3979.2 ± 102.06, *P* = 0.001). The app group (5-point Likert scale; mean score 4.37 ± 0.895) had high acceptance of the application.

**Conclusions:**

Although the app group consumed a lower PEG dose, the bowel preparation quality was similar in the two groups. Moreover, use of the smartphone camera application enhanced compliance with the bowel preparation.

## 1. Introduction

Colonoscopy is the gold standard examination for colorectal cancer screening. To guarantee an optimal adenoma detection rate, appropriate bowel preparation is essential [1]. Inadequate bowel preparation results in both failure to detect neoplastic lesions and an increased risk of procedural adverse events, as well as unnecessary short-term follow-up colonoscopy [2, 3]. Despite the importance of bowel preparation, a low bowel preparation rate was reported (20 to 25%) for all colonoscopies [4, 5].

To improve the quality of bowel preparation, many factors should be considered. Poor bowel preparation is associated with patient characteristics, such as the age, gender, health status, inpatient status, constipation, DM, use of antidepressants, history of cirrhosis, dementia, stroke, and noncompliance with cleansing instructions [6–8].

Polyethylene glycol (PEG) is one of the most common cleansing methods worldwide. In the past, a split dose of 4 L of PEG is the most preferred regimen [9, 10]. However, some previous studies [11–16] have shown that high-volume PEG (≥3 L) does not significantly increase bowel cleanliness compared to low-volume PEG (<3 L). 2 L PEG with ascorbic acid preparation is a representative low-volume PEG regimen. Studies comparing this preparation with traditional 4 L PEG preparation showed noninferior efficacy. Therefore, in healthy, nonconstipated individuals, a 4 L PEG formulation may not be superior to a lower volume PEG formulation for bowel preparation [9]. Considering this theoretical background, it is necessary to establish the optimal PEG dosage for bowel preparation in healthy individuals who lack poor preparation risk factors.

Recently, it has been reported that smartphone applications with timed alerts or checklist forms with instructions for bowel preparation have improved the quality of bowel preparation [17, 18]. We developed a novel smartphone application that helps the patient check the preparation status and optimal dose of the bowel preparation solution. The application analyzes and compares the stool status in the toilet before and after defecation. Then, the smartphone display indicates “pass or fail” for the bowel preparation. The purpose of our study was to compare the bowel preparation quality between the app and non-app groups and to assess whether the smartphone camera application could increase patient compliance with bowel preparation by individualizing the optimal dose for bowel preparation.

## 2. Materials and Methods

### 2.1. Patients

This study was conducted at a single university hospital from July to December 2014. We prospectively enrolled consecutive outpatients. Patients included men and women from 19 to 65 years of age who were scheduled for the elective colonoscopy. Patients with the following conditions were excluded: lacked a smartphone, intellectual faculties are insufficient to use smartphone application, patients with known or suspected bowel obstruction, allergy to PEG, severe chronic renal failure (creatinine clearance < 30 mL/min), pregnant or breastfeeding, presence of major psychotic illness, and did not consent to participate in the present study. All enrolled patients met with a physician to review their medical history. All patients provided written informed consent. The institutional review board approved this study and registered in the clinical trial database at https://www.clinicaltrials.gov (NCT01937819).

### 2.2. Randomization and Blinding

We generated a randomization schedule using randomly computed blocks according to the website (http://www.randomization.com). An endoscopist who did not perform the colonoscopy procedures randomly assigned patients to the app or non-app group according to the randomization schedule. The endoscopists who scored the bowel preparation and recorded the colonoscopy data were blinded to the participant information during the study period.

## 3. Bowel Preparation Protocol and Smartphone Camera Application Instructions

The preparation method using the split dose of 4 L of PEG was previously verified with an acceptable cleansing effect and tolerance [2, 10, 19]. All patients received both written and verbal instructions for colonoscopy and the importance of bowel preparation at the colonoscopy scheduling appointment. All patients were instructed to start a low-fiber diet three days before the colonoscopy. All patients had a regular diet for breakfast and lunch, which was followed by a soft diet for dinner on the day before the colonoscopy. Only clear liquids were allowed until 2 hours before the colonoscopy. The non-app group used written instructions for the colonoscopy protocol, including how to take split-dose PEG. They were instructed to take the 1st 2 L of PEG (Colyte, Taejoon Pharm Inc., Seoul, Korea; 236 g PEG, 22.74 g Na_2_SO_4_, 6.74 g NaHCO_3_, 5.86 g NaCl, and 2.97 g KCl) between 6 and 8 PM on the day before the colonoscopy and then, the 2nd 2 L of PEG approximately 6 hours before the colonoscopy.

**Figure 1:**
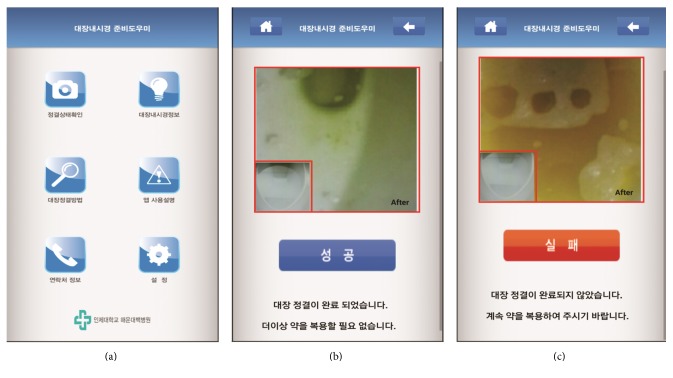
Display images of the smartphone camera application. (a) The user interface of the application, which consisted of the preparation analysis camera icon, the colonoscopy-related information icon, the bowel preparation process instruction icon, the application manual, and more. (b) The “Pass” screenshot. That image contains the message that patients can stop taking the PEG solution. (c) The “Fail” screenshot. That image contains the message that patients should keep taking the PEG solution.

For patient allocated to the app group, the smartphone camera application was illustrated by a gastroenterology fellowship doctor who did not perform the colonoscopic procedure. The android smartphone camera application consisted of the preparation analysis camera icon, the colonoscopy-related content icon, the bowel preparation process instruction icon, the application manual, and more (Figure 1(a)). The main function for it is that the user firstly gets the feces image by utilizing the camera built into a smartphone. Then, the application extracts hue, saturation, and intensity (HSI) values from the image captured and measures the feces concentration using the mean value of pixels (300 × 300) in the center part of the image. The subjects are asked to take pictures two times, before and after defecation, which helps the system compare the difference of *H* values among mean HSI values. The purpose of this application is to allow patients to determine their bowel preparation status through a simple and easy process that involves two camera shots. The smartphone camera application was programmed to automatically judge the bowel preparation conditions from the stool status in the toilet before and after defecation. The app group took the 1st 2 L of PEG on the day before colonoscopy in a manner that was similar to the non-app group. On the morning of the colonoscopy, they were supposed to check the bowel preparation status at every defecation using the application. If “Pass” was shown on display (Figure 1(b)), they stopped taking the solution. If “Fail” was shown, they were supposed to take 150 mL of the solution every 10 min (Figure 1(c)).

### 3.1. Data Collection

All patients were given a questionnaire to assess the total amount of PEG intake and the level of compliance with the instructions. Colonoscopies were performed by two endoscopists; each had experience with more than 2000 colonoscopies. Also, all colonoscopies were being done with some level of sedation (typically moderate or deep) for improving patient comfort and procedure quality. The following data were collected from each patient: age, gender, body mass index, marital status, history of abdominal or pelvic surgery, and the reason for colonoscopy.

### 3.2. Endpoints

Previous studies showed that an Ottawa bowel preparation scale (OBPS) score of less than 5 indicates adequate bowel preparation for detecting a flat adenoma [20–22]. Therefore, we set a total OBPS score of 5 as the cutoff level for satisfactory bowel preparation. The primary endpoint was the quality of bowel preparation based on blinded ratings by the OPBS score. The secondary endpoints were the difference in the PEG dosage for bowel cleansing between the two groups and acceptability of the application in the app group using the 5-point Likert scale (5-point Likert scale: 1 = unacceptable, 2 = not useful, 3 = neither useful nor not, 4 = useful, and 5 = very acceptable).

### 3.3. Statistical Analysis

Because this study is a pilot study, the number of patients in each group was determined by small sample sizes (*n* = 30). The patients' characteristics, mean OBPS scores, and PEG doses between groups were analyzed with a nonparametric rank-sum test (Mann–Whitney *U* Test). To define the acceptability of the application, Likert scale scores were also analyzed with a nonparametric rank-sum test. To assess the factors associated with good bowel preparation, all variables were entered into a univariate logistic regression model. All analyses were performed with SPSS software version 18.0 (SPSS Inc., Chicago, IL, USA). A *P* value < 0.05 was considered statistically significant.

## 4. Results

### 4.1. Baseline Characteristics

**Figure 2:**
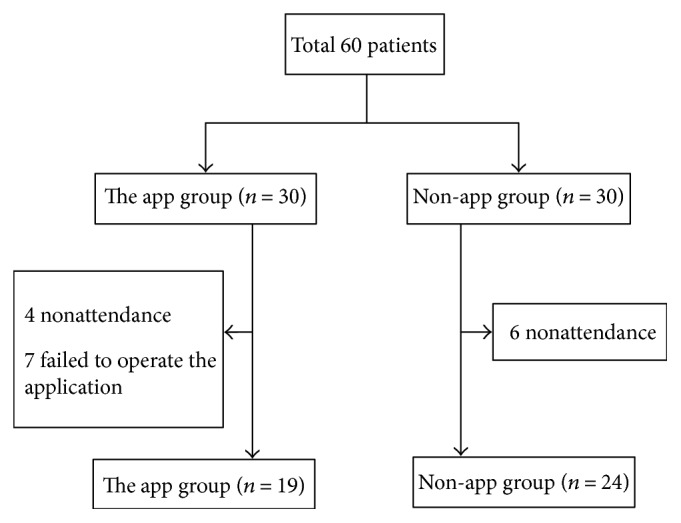
Flow diagram of the patients in the study. In the app and non-app groups, the procedure was stopped in 4 and 6 patients due to nonattendance. Seven patients in the app group failed to operate the application because they lacked an appropriate android OS version.

A total of 60 patients were enrolled in this study. Four patients in the app group and six patients in the non-app group were excluded for not undergoing a colonoscopy; seven patients in the app group were excluded because they had an android smartphone that could not operate our application. As a result, 24 patients from the non-app group and 19 patients from the app group were evaluated (Figure 2, Table 1). The patient population consisted of 56% men with a mean age of 49.4 years. Nine patients (21%) had undergone a previous abdominal or pelvic surgery. The leading indication for colonoscopy was screening (60.4%); others were symptoms (25.6%) and surveillance (13.9%). There were no differences in the gender, body mass index, marital status, history of abdominal or pelvic surgery, or indication for colonoscopy. There were no differences in the polyp and adenoma detection rates and colonoscopy withdrawal time between each group (Table 1).

### 4.2. Bowel Preparation Quality according to Application Use

**Figure 3:**
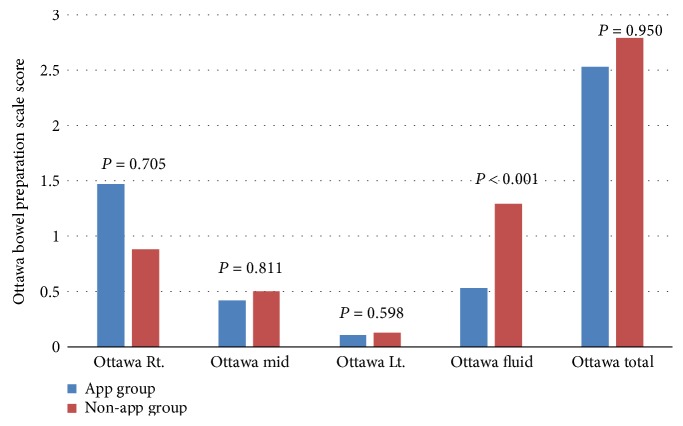
The analyses for each segment of the colon and the fluid quantity between the app and non-app groups.

The mean total score (standard deviation) of the OBPS for the 43 patients was 2.67 ± 1.74 (range 1 to 8). In a bivariate analysis of the primary outcome, a significant difference was not observed between the app and non-app groups in the bowel preparation results according to the mean OBPS scores (2.53 ± 1.264 versus 2.79 ± 2.064, *P* = 0.950). In the analyses of each colon segment and the fluid quantity, the OBPS score tended to decrease from the right to the left colon. The scores for each segment were not significantly different between the app and non-app groups. However, the score for the fluid collection was lower in the app group than in the non-app group (*P* < 0.001) (Figure 3).

### 4.3. Dosage of Purgative and Acceptability of the Application

As a result of the second outcome, the doses of PEG (mL) in the app group were significantly lower than those in the non-app group (3713.16 ± 405.81 versus 3979.17 ± 102.06, *P* = 0.001). The acceptability of the smartphone application was high (5-point Likert scale; mean score 4.37 ± 0.895) (Table 2).

### 4.4. Analyses of the Factors Associated with Good Bowel Preparation

As mentioned above, we set an OPBS score of 5 as the cutoff level for good bowel preparation after discussion with the colonoscopists participating in this study. An analysis was performed to identify any significant factors related to good bowel preparation. The factors that were analyzed included the age, gender, BMI, marital status, previous history of abdominal or pelvic surgery, PEG dose, indication for colonoscopy, and application user status. The univariate analysis did not reveal any significant variable factors (Table 3).

## 5. Discussion

To achieve optimal efficacy and sensitivity for detecting neoplastic colorectal lesions during colonoscopy, complete bowel preparation and good patient compliance are essential. Although PEG-based bowel preparation methods are safe and effective, they require ingestion of large amounts of solution for colonoscopy [23]. As a result, 5–15 percent of patients cannot complete the preparation process due to poor palatability and large volume [24, 25]. Naturally, patients prefer preparation methods that are lower in volume, more palatable, and handily consumed [2]. Therefore, several studies have attempted to improve patients' tolerance through reducing the amount of lavage solution and diminishing the volume-related symptoms, such as bloating and cramping, while maintaining efficacy. Some previous studies have been conducted to verify the efficacy of a lower dose of purgatives as an alternative PEG-based regimen [12, 23, 26, 27]. Most of these studies have shown the similar bowel cleansing effect between the low dose and standard dose groups. However, a standard dosage of PEG (4 L) has been conventionally or traditionally used without randomized controlled trial or exact evidence [10].

Recently, several methodological approaches were attempted to improve patients' compliance using education booklet and visual aids, self-help websites, short message services (SMS), or follow-up calls for reeducation for colonoscopy [21, 22, 28–30]. Another consideration is the health condition or characteristics affecting the bowel preparation quality. In many studies attempting to clarify the significant predictors of inadequate bowel preparation [1–3], inadequate preparation was related to various characteristics, such as previous inadequate bowel preparation, single and/or inpatient status, polypharmacy, obesity, advanced age, male sex, and comorbidities such as diabetes, stroke, dementia, and Parkinson's disease [2, 31, 32]. Considering these previous results, it would be expected that individual patients with predictors of poor bowel preparation need adjustments in their preparation regimens. Ultimately, it would be ideal to identify a personalized bowel preparation method using valuable interventional tools for qualified bowel preparation.

Several studies have reported on the usefulness of a smartphone application. In a study by Lorenzo-Zúñiga et al. [17], the use of a smartphone application improved the bowel preparation quality and patients' compliance through reminding patients of the bowel preparation timing or educating patients with visual aids in the application. Kavathia et al. [18] demonstrated that the use of a smartphone application assists in bowel preparation by displaying pictures of the preparation quality. Compared to these studies, we tried to ensure objectivity in the bowel preparation protocol by automating the analysis of the stool images. This approach was not only effective; it was easy to use. As a result, the app group in this study favored using this app significantly.

Nearly all current smartphones have a camera system. As a result, we developed a smartphone camera application to take stool pictures and analyze the images. During the bowel preparation process, patients execute the application and take pictures before and after each defecation. Then, the application shows the result of the bowel preparation. According to the result, the patients may stop or continue to take the cleansing agent without worrying about poor preparation. Consequently, taking the proper volume of cleansing agent could increase the patients' compliance. Moreover, some patients with risk factors for inadequate preparation were required to take larger amounts of solution, and inadequate bowel preparation can be prevented with the use of additional bowel purgatives before the exam. Thus, we applied a smartphone application that could be easily accessible and available for improving the bowel preparation quality in this study.

In this study, the app group had a significantly shorter insertion time (412.6 ± 320.0 sec versus 578.7 ± 292.9 sec, *P* = 0.013). A short insertion time decreases the total workup time, which reduces patient's discomfort. Although the app group had a shorter insertion time, the withdrawal and workup time were not significantly different between the two groups. One-step polypectomy was exercised in this study, which would influence the results of withdrawal and the total workup time. The difference in the PEG dose between the two groups is statistically significant (3713.16 ± 405.81 versus 3979.17 ± 102.06, *P* = 0.003). Additionally, the acceptability of the application in the app group showed that almost every participant rated it as 4 (acceptable) or 5 (very acceptable) points. Considering that there are no significant differences in the basal characteristics between the two groups, these results show that the patient-specific bowel purification could be easily accomplished with the use of a smartphone application.

This study has several limitations. First, it was a single tertiary center study; therefore, unexpected confounding factors could have affected the assessment of the bowel preparation quality and patient recruitment. Second, we only enrolled participants from the population of patients undergoing outpatient-based colonoscopies. Third, this application was only designed for the Android operating system (OS). As a result, it does not operate on other OS, such as iOS or Blackberry. Because many Android OS smartphones are equipped with other OS, old versions of the Android smartphone could not operate specific applications. As a result, seven patients in the app group could not use the app and were excluded from the study. This limitation will be addressed with updates to the application. Fourth, because this is a pilot study, the number of enrolled patients was small. Consequently, univariate and multivariate analyses did not show any statistically significant difference in the factors associated with good bowel preparation.

## 6. Conclusion

This novel smartphone camera application improved the bowel preparation quality and personalized bowel preparation. If we add educational contents for bowel preparation to the smartphone application, we can improve the additional cleansing effect with a visual educational program. We used the PEG solution in this study, but with this concept, it may be possible to study the use of a low level of PEG or other bowel cleansing agents. In the future, further large-scale, multicenter, randomized trials are needed to evaluate the efficacy of the smartphone camera application for optimal bowel preparation.

## Figures and Tables

**Table 1:** Baseline characteristics of the study population.

Characteristics	App group (*n* = 19)	Non-app group (*n* = 24)	Total (*n* = 43)	*P* value
Age, y	47.4 ± 8.1	51.0 ± 7.6	49.4 ± 7.9	0.103
Gender, male, number (%)	13 (68)	11 (46)	24 (56)	0.143
Body mass index, kg/m^2^	23.5 ± 2.7	22.6 ± 1.4	23.1 ± 2.1	0.471
Marital status, married, number (%)	16 (84)	22 (92)	38 (88)	0.454
Abdominal or pelvic surgery	5 (26)	4 (17)	9 (21)	0.445
Indication of colonoscopy				
Screening	11 (57.8)	15 (62.5)	26 (60.4)	0.762
Surveillance	2 (10.5)	4 (16.6)	6 (13.9)	0.568
Symptoms	6 (31.6)	5 (20.8)	11 (25.6)	0.428
Polyp detection rate (%)	31.5	41.6	37.2	0.502
Adenoma detection rate (%)	21.1	29.1	25.5	0.549
Colonoscopy withdrawal time, second	553.8 ± 322.2	579.4 ± 292.8	568.1 ± 302.7	0.599
Colonoscopy insertion time, second	412.6 ± 320.0	578.7 ± 292.9	491.1 ± 294.9	0.013
Colonoscopy work time, second	957.0 ± 439.3	1132.0 ± 379.8	1054.7 ± 411.6	0.058

Values are given as the mean ± standard deviation or number (%).

**Table 2:** Quality of the bowel preparation and PEG dosage.

	App group (*n* = 19)	Non-app group (*n* = 24)	*P* value
OBPS score, mean ± SD	2.53 ± 1.264	2.79 ± 2.064	0.950
OBPS score< 5, number (%)	18 (95)	19 (79)	0.148
Dosage of PEG (mL)	3713.16 ± 405.81	3979.17 ± 102.06	0.001

PEG: polyethylene glycol; OBPS: Ottawa bowel preparation scale.

**Table 3:** Univariate analysis of the factors associated with good bowel preparation (OBPS score< 5).

	Odds ratio	95% CI	*P* value
Age	0.921	0.823–1.031	0.1533
Gender (male : female)	0.588	0.096–3.617	0.567
Body mass index	1.143	0.723–1.809	0.567
Marital status (married)	0	0	0.999
Dosage of PEG	0.999	0.995–1.003	0.638
Application user (app group)	4.737	0.503–44.572	0.174
Abdominal or pelvic surgery	0.467	0.071–3.077	0.428
Indication of colonoscopy			
Screening	3.692	0.594–22.940	0.161
Surveillance	0.781	0.075–8.149	0.837
Symptoms	0.276	0.046–1.638	0.157
Presence of polyp	1.217	0.197–7.534	0.832
Detected adenoma	1.852	0.192–17.859	0.594

Values are given as the mean ± standard deviation or number (% or range). PEG: polyethylene glycol.
